# Application of Recombinase Polymerase Amplification with Lateral Flow for a Naked-Eye Detection of *Listeria monocytogenes* on Food Processing Surfaces

**DOI:** 10.3390/foods9091249

**Published:** 2020-09-07

**Authors:** Sarah Azinheiro, Joana Carvalho, Marta Prado, Alejandro Garrido-Maestu

**Affiliations:** 1 Food Quality and Safety Research Group, International Iberian Nanotechnology Laboratory, Av. Mestre José Veiga s/n, 4715-330 Braga, Portugal; sarah.azinheiro@inl.int (S.A.); joana.carvalho@inl.int (J.C.); marta.prado@inl.int (M.P.); 2 Department of Analytical Chemistry, Nutrition and Food Science, School of Veterinary Sciences, University of Santiago de Compostela, Campus de Lugo, 27002 Lugo, Spain

**Keywords:** RPA, lateral flow, *L. monocytogenes*, surface analysis, food processing

## Abstract

The continuous contamination of foods with *L. monocytogenes*, highlights the need for additional controls in the food industry. The verification of food processing plants is key to avoid cross-contaminations, and to assure the safety of the food products. In this study, a new methodology for the detection of *L. monocytogenes* on food contact surfaces was developed and evaluated. It combines Recombinase Polymerase Amplification (RPA) with the lateral flow (LF) naked-eye detection. Different approaches for the recovery of the bacteria from the surface, the enrichment step and downstream analysis by RPA-LF were tested and optimized. The results were compared with a standard culture-based technique and qPCR analysis. Sampling procedure with sponges was more efficient for the recovery of the bacteria than a regular swab. A 24 h enrichment in ONE broth was needed for the most sensitive detection of the pathogen. By RPA-LF, it was possible to detect 1.1 pg/µL of pure *L. monocytogenes* DNA, and the complete methodology reached a LoD_50_ of 4.2 CFU/cm^2^ and LoD_95_ of 18.2 CFU/cm^2^. These results are comparable with the culture-based methodology and qPCR. The developed approach allows for a next-day detection without complex equipment and a naked-eye visualization of the results.

## 1. Introduction

With a mortality rate of 15.6%, reported by EFSA and ECDC in 2018, listeriosis is one of the most serious food-borne diseases under EU surveillance, and since year 2000 an increase in the number of cases was observed [1]. Due to the severity of the disease, *L. monocytogenes* contamination needs to be accurately controlled. The regulation specifies “absence/25 g” in Ready-To-Eat (RTE) foods [2], and the food production line needs to be clean and safe to handle these type of products [3]. Aseptic measures are needed to avoid cross-contaminations in the processing environment [4]. However due to its ability to survive in harsh environments, such as low temperature [5], and high salt concentrations, along with its capacity to create biofilms [6], *L. monocytogenes* can persist in the food supply chain, even with regular sanitation, which may lead to contamination of food [7]. Processing plants should be tested for the presence of this pathogen to identify possible problems in the food factory and to implement corrective measures [8].

Several regulatory agencies and organization, such U.S. Food and Drug Administration (FDA), and the International Organization for Standardization (ISO) have designed standardized methodologies for the detection of *L. monocytogenes* [9,10]. In Europe, the ISO method is the one implemented in most food industries, where a primary and secondary enrichment prior to plating onto selective media is performed. These culture-based techniques are laborious and time-consuming. These techniques can take more than one week to reach the results, not being compatible with the intense production existing nowadays, as several batches may be contaminated and consumed before knowing if the product is completely safe. For this reason, molecular methodologies based on DNA analysis, such as qPCR, have been developed for the detection of pathogens allowing faster results [11,12,13,14]. Efforts have been made for the validation of molecular methods to improve the reliability and applicability of PCR/qPCR methods [15,16,17,18]. Alternative techniques like isothermal amplification have shown several advantages such as removing the necessity of complex equipment, like thermocyclers, or the possibility of automation reducing the need for highly trained personnel. All these characteristics allows to reduce time, and also the cost of the analysis [19]. Among these, the most popular one is Loop-mediated isothermal AMPlification (LAMP). Several studies have been made for the validation of this alternative technique for pathogen detection in food industries [20,21], due to its reported robustness, sensitivity and specificity [22], but the complexity of primer design and lack of fluorescent probes are among its limitations, and represent major drawbacks for certain users. More recently, Recombinase Polymerase Amplification (RPA) was developed, and has been demonstrated to overcome the limitations of LAMP assays [23]. RPA is capable of amplifying the target region in a similar way to the extension step of PCR, can take place at ambient temperature, between 37–42 °C, and the amplification may be performed in less than 10 min [24,25].

Both isothermal approaches have already been applied for the detection of different pathogens in foodstuffs [26,27,28,29,30], and allow for the possibility of a naked-eye detection in different ways, what also makes them very attractive for certain applications. Among the possibilities, the most popular ones are color change [31,32], turbidity [33] or lateral flow (LF) [33], making the interpretation of the results easier. The LF technology uses gold nanoparticles. The dipstick is designed to develop qualitative or quantitative rapid test systems for several analytes such as proteins, antibodies, or gene amplifications. The probe contains a first detector labelled with FAM or FITC, and the reverse primer a second labelled with biotin. The RPA amplified fragment will be then labelled with both molecules (FAM and biotin), it is loaded in then the dipstick, and placed into a buffer. The amplified fragment, labelled with FAM and biotin, binds first to the gold-labeled FAM-specific antibodies in the sample application area of the dipstick. By capillarity the gold complexes diffuse over the membrane. Only the analyte captured gold particles will bound when they overflow the immobilized biotinligand molecules at the test band and generate there a red-blue band over the time. Not-captured gold particles flow over the control band and will be fixed there by species-specific antibodies. With increasing incubation time, the formation of an intensely colored control band appears (https://www.milenia-biotec.com/en/product/hybridetect/).

In this study, we aimed to develop a novel method combining RPA amplification with LF naked-eye detection, for the rapid assessment of *L. monocytogenes* contamination on food processing surfaces. The methodology was compared against another molecular method (qPCR) and the European reference culture-based method for *L. monocytogenes*, ISO 11290-1.

## 2. Materials and Methods

### 2.1. Pure Culture Preparation

Pure cultures of different bacterial strains were prepared by the inoculation of one isolated colony in 4 mL of Nutrient Broth (NB, Biokar Diagnostics S.A., Allonne, France) which were incubated overnight (ON) at 37 °C. All strain used in this study are listed in Table 1.

For the surfaces contamination experiments, *L. monocytogenes* WDCM 00021 was selected as the reference strain, and used in all experiments. The fresh ON culture was ten-fold serially diluted in NB, and the different dilutions were used to spike the desired bacterial concentration on the surfaces. Two consecutive dilutions were plated on Tryptone Soya Yeast Extract Agar (TSYEA, Biokar Diagnostics S.A., Allonne, France), the plates were incubated at 37 °C ON to determine the exact amount of viable bacteria inoculated.

### 2.2. RPA Primers Design

Two different previously published sets of primers were tested to evaluate which one provided the best results in terms of sensitivity and specificity by the RPA assay, in combination with a novel LF probe. Both sets targeted the *hlyA* gene. One set of primers was previously designed for qPCR, tested and evaluated in several studies for this purpose [24,34]. The other set of primers was specifically developed for RPA following the recommendation of TwistDx (TwistDX, Maidenhead, UK), being these longer than the other primers. These primers were designed and evaluated in previous studies for the qRPA detection of *L. monocytogenes* [24,35], and were modified in this study, labelling the reverse primer with biotin on 5′ end to allow the lateral flow detection. The LF probe was also designed following TwistDX guidelines, with 5′ FAM, an internal tetrahydrofuran residue (THF) and a C3 spacer (SpC3) on the 3′ end. The detailed sequence of each primer and probe used with their specific modifications are presented in Table 2.

### 2.3. Surface Contamination and Sampling

Stainless steel coupons of 100 cm^2^ (10 cm × 10 cm) were used to mimic food processing plants. Before use, the surfaces were sterilized (autoclaved at 121 °C for 15 min) and cleaned (air dried, cleaned with 10% bleach and 70% ethanol and finally exposed to UV light for 15 min). After the sterilization process was completed, 100 µL of the selected dilution, prepared as described in Section 2.1, was added to the surface in droplets and uniformly spread on the coupons until dry. The inoculated surfaces were incubated 30 min at room temperature (RT, Moscow, Russia) before sampling. For the recovery of the bacteria, two different tools were tested, a Rayon Sterilin™ Plain Swab (Thermo Fisher Scientific Inc., Waltham, MA, USA) and 3M™ Sponge-Sticks (3M, Saint Paul, MN, USA). The surface was scratched 10 times in each direction (horizontal, vertical, and diagonal) with the swab or sponge pre-moistened with 0.01% Tween 80 in Phosphate-Buffered Saline (PBS, 137 mM NaCl, 12 mM Phosphate, 2.7 mM KCl, pH 7.4). The swab/sponge was introduced in 3 mL of a selective medium for *L. monocytogenes* (detailed below)*,* homogenized manually, and incubated for 24 h at 30 °C.

### 2.4. Optimization of Enrichment Step

ONE broth (OXOID, Hampshire, UK) and Half Fraser (HF, Biokar Diagnostics S.A., Allonne, France) were compared to determine which was most suitable for the enrichment of *L. monocytogenes*. To do so, 200 µL of the media were inoculated with 2 µL of fresh bacterial culture diluted to a final concentration of 10^2^–10^3^ CFU/mL. The growth of the pathogen was monitored for 24 h, at 30 °C, measuring the optical density at 600 nm (OD600) every 30 min in a Microplate Reader (Synergy, BioteK H 1, Winooski, VT, USA), with constant agitation. Different parameters were evaluated, such as the duration of the lag phase (λ), maximum optical density (ODmax), and the maximum specific growth rate (µmax) as previously described [24].

The time of enrichment was also evaluated to understand the effect on the Limit of Detection (LoD). A 2 mL aliquot was taken right after sampling (without enrichment six samples were analyzed, three inoculated with 10^4^ CFU/cm^2^ and another three with 10^3^ CFU/cm^2^) or after 8 h and 14 h. For each time point three samples were analyzed, spiked with 10^4^ CFU/cm^2^, 10^3^ CFU/cm^2^ and 10^2^ CFU/cm^2^. The results obtained were compared to those of a 24 h enrichment. The DNA extraction was conducted as described in Section 2.5.2

### 2.5. DNA Extraction

#### 2.5.1. Pure Culture Cell Lysis

From the ON pure culture cell lysates were performed to test the inclusivity and exclusivity of the RPA reaction. One mL of the culture was centrifuged for 5 min at 16,000× *g*, the pellet was washed with 1 mL of TE 1× (10 mM Tris-HCl, 1mM EDTA, pH 7.5) and centrifuged again in the same conditions. The supernatant was discarded and the pellet res-suspended in 300 µL of TE 1×. The aliquots were incubated at 99 °C for 15 min with constant agitation at 1400 rpm. Finally, were centrifuged at 16,000× *g* for 5 min at 4 °C to separate the DNA from the cellular debris. The lysates were quantified in a NanoDrop 2000c (Thermo Fisher Scientific Inc., Waltham, MA, USA), and stored at −20 °C until needed.

#### 2.5.2. Surface Samples

After the enrichment step, 2 mL of the sample was centrifuged at 16,000× *g*, during 5 min, to recover the bacterial cells, the pellets were washed with 1 mL of PBS and centrifuged again. The DNA extraction was performed with the NucleoSpin^®^ Tissue kit (Macherey-Nagel, GmbH & Co KG, Düren, Germany), using the support protocol established for bacteria with some modifications. Briefly, after the washing with PBS, the pellet was resuspended in 200 µL of the enzymatic solution containing 1 mg/mL of achromopeptidase and 20 mg/mL of lysozyme in TE 2X with 1.2% of Triton X-100. Also 25 µL of proteinase K (Macherey-Nagel, GmbH & Co KG, Düren, Germany) were added, and the suspension was incubated at 37 °C for 30 min. Then the protocol was followed as described by the manufacturer, continuing from step 3 with the addition of 200 μL of Buffer B3. The elution was modified, by being performed in two times, adding 50 μL in each time. The DNA extracts were stored at 4 °C until use.

### 2.6. Food Contact Surface Sample Analysis

The samples were analysed by three different approaches. The developed methodology with RPA-LF, was compared against qPCR and the culture-based method.

#### 2.6.1. RPA-LF

The RPA reactions were performed in a Veriti Thermal Cycler (Applied Biosystems™, Foster City, CA, USA) using the primers designed for qPCR (*hly*-P3F/*hly*-P3R) in a concentration of 420 nM and 120 nM of the LF probe (*hly*-LF-P). The reactions were performed in a final volume of 25 µL with 2 µL of the template using the TwistAmp^®^ nfo kit (TwistDX, Maidenhead, UK), following the manufacturer recommendations. The amplification was performed at 39 °C for 40 min.

Once the amplification reaction was completed, the LF was done using Milenia^®^ HybriDetect universal test strip (dipstick). To do so, a 1/50 dilution in HybriDetect Assay Buffer (Milenia Biotec GmbH, Giessen, Germany) or PBS was done, and 10 µL were loaded directly on the sample application area of the LF strips (Milenia Biotec GmbH, Giessen, Germany). The strips were introduced in a clean 1.5 mL tube containing 100 µL of the same buffer used for the dilution, and incubated for 5 min until the control band was clearly visible.

#### 2.6.2. qPCR

The results obtained by RPA-LF analysis were compared to qPCR. The method was developed in a previous study, where a competitive internal amplification control (IAC) was also incorporated to identify false negative results due to reaction inhibition [24,34]. The sequences of the primers and probes are listed in Table 2. The concentration used were: 200 nM of *hly*-P3F and *hly*-P3R, 150 nM of *hly*-P3P (probe), 100 nM of IAC-P3 probe and 2000 copies of IAC DNA. The reaction was performed in the final volume of 20 µL with 3 µL of DNA template, using 10 µL of TaqMan™ Fast Advanced Master Mix (Applied Biosystems™, Foster City, CA, USA) in a QuantStudio^TM^ 5 System and analysed with QuantStudio^TM^ Design & Analysis Software v1.5.1 (Applied Biosystems™, Foster City, CA, USA). The fast thermal profile recommended by the manufacturer was selected, only modifying the annealing/extension temperature. First, a Uracil-DNA Glycosylase (UDG) treatment was performed (2 min at 50 °C), this was followed by a hot-start polymerase activation at 95 °C for 2 min, and finally 40 cycles of denaturation at 95 °C during 1 s and the annealing/extension at 63 °C for 20 s.

#### 2.6.3. Culture-Based Methodology

To confirm the results obtained by the DNA-based method, a traditional approach, based on the ISO 11290-1 was performed. After an enrichment step of 24 h in ONE broth, a loopful was streaked on COMPASS Listeria plates (COMPASS, Biokar Diagnostics S.A., Allonne, France). The growth of typical colonies was observed after 24 h at 37 °C. Additionally, 100 µL of enrichment medium were transfer to 10 mL of Full Fraser broth (FF, Biokar Diagnostics S.A., Allonne, France) and observed if the medium turned dark after 24–48 h at 37 °C, and streaked on COMPASS plates to confirm the presence of *L. monocytogenes* (the plates were incubated under the same conditions detailed above).

### 2.7. Evaluation

#### 2.7.1. RPA-LF Evaluation

The inclusivity/exclusivity and dynamic range of the RPA-LF were evaluated testing both sets of primers. Forty two different strains were analyzed (Table 1). For this purpose DNA of cell lysates from an ON fresh culture of each strain were used as the template in the RPA reaction and results were observed after loading the amplified product in the LF strips, as described in Section 2.6.1

For the evaluation of the dynamic range two different approaches were followed, to determine the lowest DNA concentration and bacterial concentration need to have a positive result by RPA-LF. For the first approach, ten-fold serial dilutions were made from a *L. monocytogenes* cell lysate obtained as described in Section 2.5.1 and used as a template in the RPA reaction. For the second one, ten-fold serial dilutions of an ON pure culture were done and the cell lysates were prepared from each one of this dilutions also as described in Section 2.1 and Section 2.5.1.

#### 2.7.2. Methodology Evaluation

For the evaluation of the full methodology a total of 32 surface samples were tested, with different contamination levels, ranging from 10^5^ to 1 CFU/cm^2^ (Table 3). The limit of detection (LoD) was calculate using PODLOD calculation program, version 9 [30] to predict the Probability of Detection (POD) of *L. monocytogenes* on the surface using the developed methodology in different conditions. The identification of Positive and Negative Agreement (PA/NA) when the alternative methodology present the same results as the reference; False Negative (FN) and False Positive (FP) when the alternative methodology gives a different result from the reference, and the confirmation in COMPASS is in concordance with the reference; and True Positive (TP) when the reference methodology give a negative result and the alternative methodology and confirmation shows to be positive. This values allowed to calculate the relative specificity (SP), sensitivity (SE) and accuracy (AC), observed and expected frequency of agreement (p_0_/p_e_) and the Cohen’s kappa (*k*). The validation of the technical parameters has been performed according to the NordVal guidelines [36].

## 3. Results

### 3.1. Evaluation of RPA-LF Reaction and Comparison of Primers

#### 3.1.1. Dynamic Range of RPA-LF

**Figure 1:**
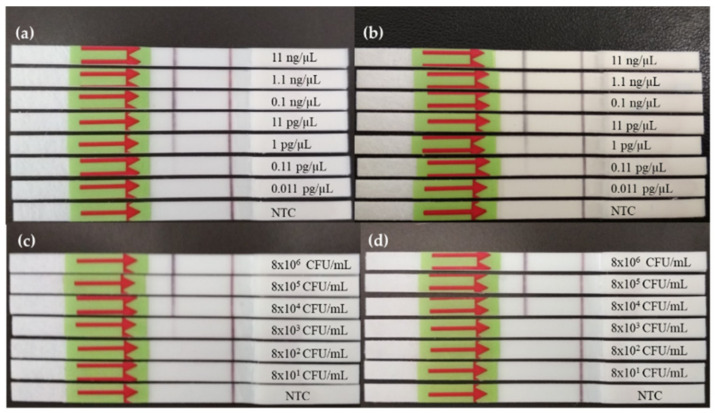
Dynamic range of *L. monocytogenes* detection for RPA-LF reaction. (**a**) and (**b**) show the lowest concentration of DNA possible to detect with qPCR and RPA primers, respectively; (**c**) and (**d**) present the results of the lowest bacteria concentration detected by RPA-LF using qPCR and RPA primers, respectively. NTC: non-template control.

To determine the best primer set to be used with the RPA in combination with LF detection, first the lowest concentration of a *L. monocytogenes* DNA with each set, yielding a positive result was determined; and second, the concentration of *L. monocytogenes* cells needed to have a positive result by RPA-LF was also determined, this was performed by preparing ten-fold dilutions from an ON culture and preparing cell lysates from each dilution. Both sets of primers obtained comparable results. As depicted in Figure 1a,b, it was possible to reliably detect down to 1 pg/µL. The use of qPCR primers seemed to generate slightly less intense bands. When the evaluation was focused on bacterial cultures, it was observed that a concentration of 8 × 10^4^ CFU/mL was needed with both sets of primers for reproducible detection, as shown in Figure 1c,d.

#### 3.1.2. Inclusivity/Exclusivity

**Figure 2:**
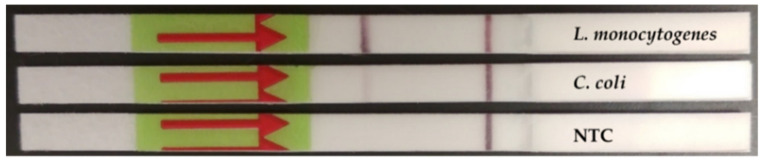
Lateral flow strips showing the difference in the intensity of the test band with a specific (*L. monocytogenes*) and non-specific (*C. coli*) detection obtained with RPA amplification, using PCR primers. NTC: non-template control.

The DNA from a total of 42 cell lysates from the strains detailed in Table 1, and obtained as described in Section 2.5.1, were tested to evaluate the inclusivity and exclusivity of the RPA-LF assay. For the inclusivity, a total of 11 *L. monocytogenes* strains were analysed, while for the exclusivity 7 other *Listeria* spp., and 24 different bacterial strains were screened. The results of the evaluation of the inclusivity and exclusivity are presented in Table 1. All *L. monocytogenes* strains were correctly identified by RPA-LF. Faint bands, as exemplified in Figure 2, were observed in 4 strains for each set of primers. These results allowed us to establish a threshold in the intensity of the band to consider a positive result.

### 3.2. Sample Pre-Treatments Optimization

To reach the lowest limit of detection, the full methodology was optimized, to improve the recovery of the bacterial cells from the surface, and the enrichment step.

#### 3.2.1. Sampling Procedure

**Figure 3:**
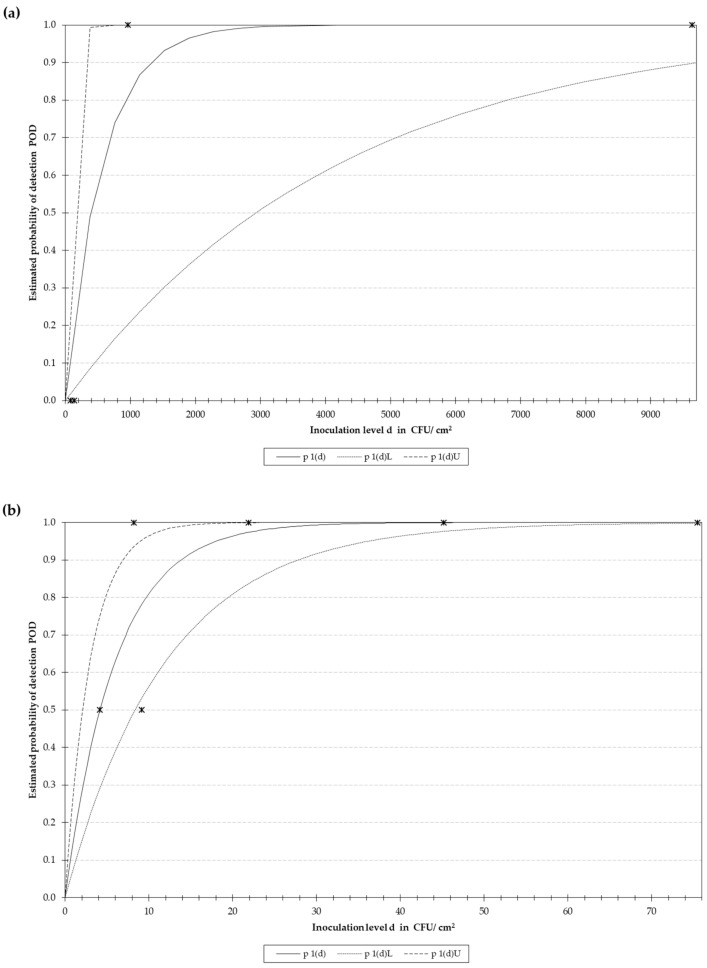
Optimization of sample pre-treatment. Two approaches for sampling were tested. (**a**) represents the estimated probability of detection (POD) using a swab and (**b**) using a sponge. “p 1(d)” represents the POD, “p 1(d)L” and “p 1(d)U” the Lower and Upper limit and the 95% confidence band, respectively, and the “*” indicate the actual values obtained for the samples analyzed included in the model. The results were obtained after a 24 h enrichment in ONE broth, and analysis with the primer set *hly*-RPA F/*hly*-RPA R. Note that in Figure 3a the lower limit does not reach the maximum as the X-axis was cut before 10 × 10^3^ CFU/cm^2^ for a more clear visualization of the results, the missing sample was inoculated with a concentration of 75 × 10^3^ CFU/cm^2^.

Two different approaches were compared (swab and sponge) to determine the optimal procedure to recover *L. monocytogenes*. Both tools were used the same way (moistened device, same sampling surface, number of times and direction the devices were passed) and after enrichment, the limit of detection by RPA-LF was evaluated. The swab reached a LoD_50_ of 391 CFU/cm^2^ (95% C.I.: 52.3–2930.6 CFU/cm^2^), while for the sponge it was calculated to be 4.2 CFU/cm^2^ (95% C.I.: 2.1–8.5 CFU/cm^2^). Must be noted that these results differ from those presented for the dynamic range as these data were generated after sample enrichment. In Figure 3a,b the POD is depicted, including the 95% confidence interval, showing that the sponge allowed to improve the recovery of the bacterial cells from the surface by 2 logs, compared to the swab.

#### 3.2.2. Enrichment Broth

**Figure 4:**
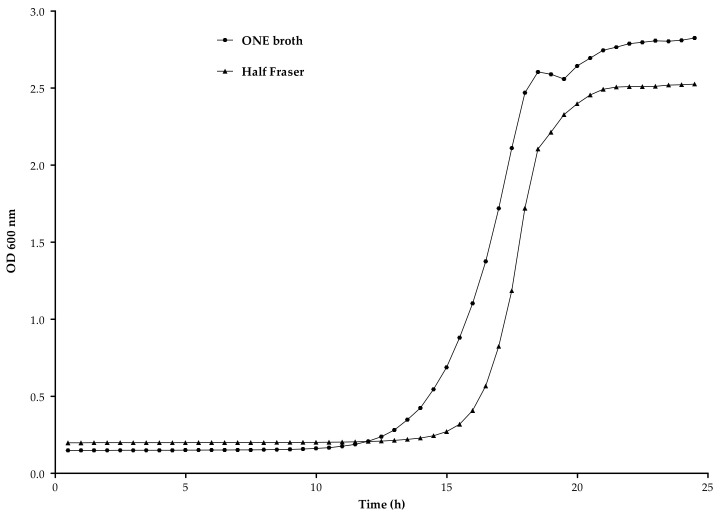
Comparison of *L. monocytogenes* growth kinetics in Half Fraser and ONE broth medium. Optical Density (OD) read at 600nm, every 30 min, during 24 h at 30 °C with constant shaking.

A kinetic analysis of the growth of *L. monocytogenes* was performed to evaluate two different media, Half-Fraser and ONE broth, to improve the enrichment step. Figure 4 depicts the growth curves obtained with both media. Half Fraser showed a λ, ODmax and µmax of 16.25 ± 0.3, 2.33 ± 0.04 and 0.90 ± 0.06, respectively, while ONE broth had a lower µmax (0.64 ± 0.01) but allowed to achieve a higher ODmax (2.71 ± 0.03), reducing the λ (14.28 ± 0.25). These results indicated that ONE broth outperformed HF, allowing to obtain a higher concentration of *L. monocytogenes* after 24 h of enrichment.

#### 3.2.3. Enrichment Time

**Figure 5:**
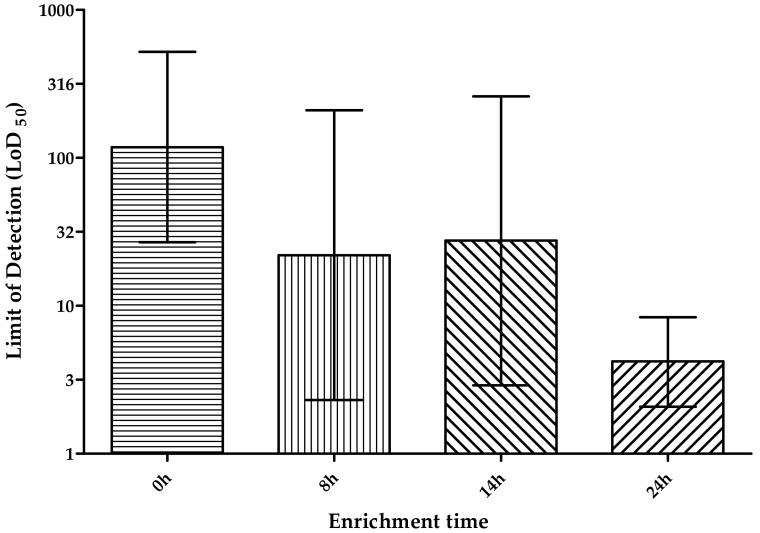
Comparison of the LoD_50_ with the 95% confidence upper and lower limits, obtained with different enrichment times (0, 8 h, 14 h, 24 h) in ONE broth. LoD_50_ calculated with PODLOD software and represented in CFU/cm^2^.

After the medium for the enrichment was selected, the time of incubation was also evaluated and the LoD at different times was determined. The LoD_50_ values obtained were 117, 21, 27 and 4 CFU/cm^2^ without enrichment, 8 h, 14 h and 24 h of enrichment in ONE broth, respectively. In Figure 5 the difference between these results is shown, highlighting the reduction of the LoD by almost 2 log when a 24 h enrichment was performed, compared with the value obtained without enrichment. A reduction of almost 1 log is observed with enrichment for 8 h and 14 h, providing these two a similar LoD. The LoD_50_ and LoD_95_ of the RPA-LF methodology with and without enrichment were compared and summarized in Table 4, presenting a LoD_95_ of 509.3 and 18.2 CFU/cm^2^ respectively.

### 3.3. Evaluation of the Methodology

The methodology combining a sampling step with the sponge, and a 24 h enrichment in ONE broth was deemed the most suitable, and the results obtained with RPA-LF were compared against the other approaches (qPCR and the culture-based as the reference). The results are summarized in Table 3. Overall, similar results were obtained with the novel RPA-LF and qPCR, and both obtained values higher than 90% for most parameters evaluated. Only two deviations were observed, one for each methodology. Regarding qPCR one FN result was obtained, and was tracked to a sample very close to the calculated LoD_50_, while for the RPA-LF one FP was observed, and corresponded to a sample positive which was negative by qPCR and the culture-based method. Most importantly, both molecular approaches obtained values higher than 0.9 in the Cohen’s k. These results are summarized in Table 5.

## 4. Discussion

The RPA-LF approach showed excellent performance, achieving similar results to those of the qPCR methodology, obtaining a LoD_50_ of 4.2 CFU/cm^2^, which is comparable with other approaches developed for the detection of *L. monocytogenes* on surfaces. Some different alternatives have been recently developed for this purpose [37,38], but to the best of our knowledge, this is the first study applying RPA combined with LF for food contact surface analysis. This type of methodology has been previously applied in food samples [33,39], but no prior studies have been identified reporting its application to food-contact surfaces.

The methodology showed some slight cross-reactivity with other bacterial species during the evaluation of the inclusivity and exclusivity, as a faint band in the LF strip was visible. It was previously reported that extended incubation times while performing the LF could lead to the appearance of faint bands [40], in the same way previous studies have indicated that by applying a higher dilution factor could solve this issue [41]. It was also recently reported that faint bands may be visible in LF assays linked to the structure and size of the probes, which may not be related with cross-reactivity [42], this seems like a feasible explanation of the results observed as the lack of cross-reactivity has been described previously using the same probe but in exo RPA [24,30]. As commented above, this was not considered an issue as these results served to determine a band intensity threshold, and so to avoid any possible sample misidentification.

It was observed that the limit of detection was not restricted by the detection methodology (RPA-LF, qPCR, or culture-based) but by the sampling process, as this step limited the number of bacteria recovered from the surface. Several studies have reported bacterial loses in the sampling step, the influence of the tool employed for the sampling procedure, as well as the type of surface tested [43,44]. Sponges are normally preferable in bigger areas, and in this study the use of this tool allowed to improve the sensitivity of the methodology by almost 2 log CFU/cm^2^, compared to cotton swabs. The results obtained are in agreement with previous studies [43]. The higher contact area of the sponge, and the robustness of the stick, allowed for a stronger rubbing of the surface and the recovery of more bacteria than the swab, which was smaller and more fragile. Swabs have been reported to be more appropriate to be used in hard-to-reach small areas [45]. In this study, after inoculation of the surfaces, the bacteria were air dried and recovered 30 min later in an attempt to mimic more closely what happens in the food industry. This process can hinder the recovery of the bacteria, as different studies reported the ability of this pathogen to attach quickly, in less than 20 min, to different food contact materials [46], showing a reduction of 2 log CFU in stainless steel surfaces after 30 min [47]. Dry surfaces also increase de adhesion of the bacteria, that is also why the European guidelines recommend the use of moistened wiping devices for the recovering. The degree of pressure applied on the swabbing device also influences the recovery of bacteria, which can affect the repeatability of the method, producing different results in samples contaminated with the same bacterial concentrations [43]. These facts may explain why, even though a sample inoculated with 20 CFU/cm^2^, which is above the LoD_95_ of the RPA-LF (18 CFU/cm^2^), obtained a negative result. Also, it worth to mention that even though both samples were analyzed using the same DNA extract, this was not considered as a ND by qPCR because the calculated LoD_95_ for this particular technique was higher than the inoculation level (36 CFU/cm^2^).

As expected, the implementation of a 24 h enrichment step, allowed to significantly reduce the LoD. In this sense, the possibility to improve the time of analysis by the reduction of the enrichment was also evaluated. A time reduction to 8 h or 14 h increased the LoD of 1 logarithmic unit. The evaluation of the growth rates was performed the reference strain recommended by the ISO 11133 for the evaluation of culture media (WDCM 00021), and variations in this rate may be observed with other strains. In RTE products, where absence of *L. monocytogenes* is required, it is essential to ensure that the food processing plants are clean to avoid cross-contamination. For this reason the 24 h enrichment was selected for the final RPA-LF methodology. The length of this step is comparable to the time reported in other studies dealing with the detection of *L. monocytogenes* when applying isothermal [35,48] or other molecular techniques [34,49,50]. The results obtained for the different parameters evaluated (SE, SP, AC, and *k*) were similar to those previously reported applying other molecular methods targeting *L. monocytogenes*. The high values obtained in these parameters (≥89%) along with the positive comparison versus previously published studies, confirms the good performance of the novel method [30,35]. Furhtermore, the values obtained for SE and *k* fulfill the requirements of the NordVal regulation for alternative methods, as the values obtained were higher than 95% and 0.80 respectively, thus demonstrating the reliability of the novel assay [36].

The developed approach is less time consuming, compared with the traditional culture-based methods, allowing a next-day detection, and does not need specialized personnel or equipment as the RPA-LF does not need complex equipment for the amplification step, and the naked-eye detection simplifies the assessment of positive/negative samples removing any kind of complicated analysis for the results interpretation.

## 5. Conclusions

A novel methodology based on isothermal DNA amplification (RPA) was developed and combined with instrumentation-free detection by combining it with LF strip. The assay was tested in stainless steel coupons to mimic food contact surfaces from a processing facility, and highly sensitive results were obtained when a 24 h enrichment step was included. The results were comparable to those of qPCR, and even a culture-based approach. Overall, the novel methodology has great potential for its implementation in the food industry to improve the current culture-based testing methodologies.

## Figures and Tables

**Table 1:** List of bacterial strains used, and results obtained, to evaluate the inclusivity/exclusivity of two sets of primers.

Bacteria Species	Source	N	qPCR Primers	RPA Primers
*Salmonella* spp.	Mollusk (AMC 90, 238, 253, 255)	4	-	-* 238
*Salmonella* spp.	Unknown (AMC 260, 261)	2	-* 261	-
*Salmonella* Oranienburg	Mollusk (AMC 28)	1	-	-
*Salmonella* Veneziana	Mollusk (AMC 200)	1	-	-
*Salmonella* Anatum	Proficiency test (AMC 60)	1	-	-
*Salmonella* Enteritidis	Proficiency test (AMC 82)	1	-	-
*Salmonella* Enteritidis	University of Bristol	1	-	-
*Salmonella* Wentworth	Proficiency test (AMC 84)	1	-*	-
*Salmonella* Wentworth	Mollusk (AMC 200)	1	-	-
*Salmonella* Liverpool	Proficiency test (AMC 198)	1	-	-*
*Salmonella* Typhimurium	Proficiency test (AMC 96)	1	-	-
*Salmonella* Typhimurium	Unknown (WDCM 00031)	1	-	-
*Listeria monocytogenes*	Spinal fluid, serotype 4b (WDCM 00021)	1	+	+
*Listeria monocytogenes*	Mollusk	4	+	+
*Listeria monocytogenes*	Chestnut	3	+	+
*Listeria monocytogenes*	Chicken	3	+	+
*Listeria ivanovii*	WDCM 00018	1	-	-
*Listeria innocua*	WDCM 00017, CECT 5376, 4030, 1325, 1141, 2110	6	-* 1141	-
*Staphylococcus aureus*	WDCM 00034, 00033	2	-	-* 00033
*Staphylococcus* coagulase positive	Proficiency test	1	-	-
*Campylobacter coli*	University of Minho	1	-*	-*
*Escherichia coli*	WDCM 00013, 00012	2	-	-
*Escherichia coli*	WDCM 00014	1	-	-
*Enterococcus faecalis*	WDCM 00009	1	-	-

N: number of strains; the inclusivity and exclusivity were tested for both sets of primers for the RPA amplification and detection by Lateral Flow (LF) using TwistAmp^®^ nfo and Milenia HybriDetect 1 strips. Positive results were observed when two bands appeared in the strips, the control band and the result band. * strain number where a faint band was observed in the test line. “-” Indicates a negative result in the corresponding methodology.

**Table 2:** Primer used in RPA and qPCR reaction.

Methodology		Sequence 5′-3′	Modifications	Reference
RPA	*hly*-RPA F (forward primer)	TTACACTTATATTAGTTAGTCTACCAATTGCG	-	[35]
	*hly*-RPA R (reverse primer)	TCCAATCCTTGTATATACTTATCGATTTCATC	5′-Biotin/	
	*hly*-P3F (forward primer)	GCAACAAACTGAAGCAAAGGAT	-	[34]
	*hly*-P3R (reverse primer)	CGATTGGCGTCTTAGGACTTGC	5′-Biotin/	
	*hly*-LF- P (lateral flow probe)	TCTGCATTCAATAAAGAAAATTCAATTTCATCZATGGCACCACCAGCATC	5′-FAM/THF/SpC3 *-3′	
qPCR	*hly*-P3P (*hly* probe)	CATGGCACC//ACCAGCATCTCCG	5′-FAM/ZEN/IABkFQ-3′	[34]
	IAC-P3P (IAC probe)	AGTGGCGGT//GACACTGTTGACCT	5′-YY/ZEN/IABkFQ-3′	
	IAC-DNA	GGATTACCCTAGAGTGGCGGTGACACTGTTGACCTTCTATTACCTC		

*hly*-P3F and *hly*-P3R are the same primer sequence used in qPCR methodology. For the RPA-LF assay, the *hly*-P3R was modified with a biotin on 5′. ”*” indicates the position of the THF.

**Table 3:** Surface samples analysis by RPA-LF, qPCR and culture-based approaches.

Contamination Level (CFU/cm^2^)	*N*	RPA-LF	qPCR	COMPASS	Full Fraser 24 h	Full Fraser 48 h
8 × 10^5^	2	+	+	+	+	+
8 × 10^4^	2	+	+	+	+	+
8 × 10^3^	2	+	+	+	+	+
8 × 10^2^	2	+	+	+	+	+
8 × 10^1^	1	+	+	+	+	+
^**^ 7 × 10^1^	1	+	+	+	+	+
^**^ 5 × 10^1^	3	+	+	+	+	+
^**^ 2 × 10^1^	2	+	+	+	+	+
	1	+	-	-	-	-
^**^ 9	2	+	+	+	+	+
	2	-	-	-	-	-
^**^ 8	2	+	-	+/- *	+	+
	2	+	+	+	+	+
7	1	-	-	-	-	-
^**^ 4	2	+	+	+	+	+
	3	-	-	-	-	-
1	1	-	-	-	-	-
1	1	-	-	-	-	-

* Out of the 2 samples, 1 was positive and the other negative. Contamination level refers to the concentration of bacteria (CFU/cm^2^) inoculated in the surface by cm^2^ before enrichment in ONE broth. The samples were analysed by RPA-LF and qPCR and the results were compared with the reference culture-based methodology. “+” in “COMPASS” refers to the presence of typical colonies observed after enrichment in ONE broth (molecular method), and “+” in “Fraser” observed when media turned dark and typical colonies were observed after plating on COMPASS. “-” Indicates a negative result in the corresponding methodology. N corresponds to the number of samples analysed with the respective bacterial concentration. ^**^ Denotes the samples used for the determination of the LoD.

**Table 4:** Limit of Detection of *L. monocytogenes* with developed methodology and qPCR.

Methodology	LoD_50_ *			LoD_95_ *		
	LoD	Lower Conf. Limit	Upper Conf. Limit	LoD	Lower Conf. Limit	Upper Conf. Limit
RPA-LF without enrichment	117.9	26.9	516.0	509.3	116.3	2230.0
RPA-LF with enrichment	4.2	2.1	8.5	18.2	9.1	36.5
qPCR	8.3	4.1	16.8	36.1	17.9	72.5

* CFU/cm^2^. qPCR methodology was accomplished with a 24 h enrichment in ONE broth to be compared with RPA-LF.

**Table 5:** Evaluation of the results.

Approach	N	PA	NA	FN	TP	FP	AC (%)	SE (%)	SP (%)	p_0_	p_e_	*k*
qPCR	32	21	10	1	0	0	97	95	100.0	0.97	0.44	0.94
RPA-LF	32	23	8	0	0	1	97	100	89	0.97	0.40	0.95

N: number of samples, PA: positive agreement, NA: negative agreement, FN: false negative, TP: true positive, FP: false positive, AC: relative accuracy, SE: relative sensitivity, SP: relative specificity, p_0_: proportion of agreement, p_e_: expected frequency of agreement, *k*: Cohen’s kappa.
